# Microsurgical resection of a large cavernous malformation of the medulla oblongata

**DOI:** 10.3171/2019.7.FocusVid.1998

**Published:** 2019-07-01

**Authors:** Sima Sayyahmelli, Mustafa K. Başkaya

**Affiliations:** Department of Neurological Surgery, University of Wisconsin–Madison School of Medicine and Public Health, Madison, Wisconsin

**Keywords:** cavernous malformation, developmental venous malformation, medulla oblongata, microsurgical resection, midline suboccipital craniotomy, video

## Abstract

In this surgical video, we present a 57-year-old man with neck pain, dizziness, and imbalance. MRI showed a heterogeneously enhancing mass lesion within the posterior medulla at the level of the foramen magnum. Because the patient was symptomatic from this cavernous malformation, the decision was made to proceed with surgical resection. The patient underwent a midline suboccipital craniotomy with C1 laminectomy for surgical resection of the cavernous malformation in the medulla oblongata, with concurrent monitoring of motor and somatosensory evoked potentials.

The surgery and postoperative course were uneventful. The postoperative MRI showed gross-total resection of the mass with histopathology indicating a cavernous malformation. The patient continues to do well without recurrence at 7 years of follow-up. In this video, we demonstrate important microsurgical steps for the resection of this challenging and rare vascular malformation.

The video can be found here: https://youtu.be/gbGleLowzxo.

Surgical resection of cavernous malformations involving the medulla oblongata can be more challenging than other brainstem locations. This is due to the presence of densely packed vital neural structures in the medulla oblongata.

## Transcript

In this video we demonstrate important steps in the microsurgical resection of a large, challenging cavernous malformation in the medulla oblongata.

The patient is a 57-year-old man with neck pain, dizziness, and imbalance **(0:30)**. MRI showed a heterogeneous enhancing intra-axial mass lesion located within the posterior medulla at the level of the foramen magnum **(0:34)**. Because the patient was symptomatic from this cavernous malformation, the decision was made to proceed with surgical resection. The patient underwent a midline suboccipital craniotomy with C1 laminectomy **(0:43)** for surgical resection of the cavernous malformation in the medulla oblongata, with concurrent monitoring of motor and somatosensory evoked potentials.

The patient was placed in the prone position **(1:01)**. A midline skin incision was marked. A midline suboccipital craniotomy was then performed by placing two burr holes on either side of the occipital keel **(1:05)**. Since the lesion extended below the level of the posterior foramen magnum, the posterior arch of the C1 was also removed. We then opened the dura. At this time point, baseline neuromonitoring responses of both somatosensory and motor evoked potentials were obtained, which were then continuously monitored throughout the remainder of the case **(1:18)**.

The arachnoid of the cisterna magna was opened sharply with the aid of microscope magnification and illumination **(1:29)**. Upon opening the thick arachnoid, a large vascular malformation with the typical appearance of cavernous malformation came to view at the lower aspect of the medulla oblongata **(1:35)**. This lesion was large and expanded the medulla oblongata. The right posterior inferior cerebellar artery was observed. The loop of PICA was then dissected and freed from the cavernous malformation **(1:40)** and kept in place using a Telfa patty. An unusually large developmental venous malformation with multiple tributaries was also observed on the dorsal surface of the malformation **(1:44)**. This DVA was dissected away with spreading motions of the bipolar forceps and untethered from arachnoid bands. During dissection, we encountered an orange color fluid coming from around the malformation, indicating hemosiderin from an old hemorrhage **(2:23)**. We then used gentle bipolar coagulation to shrink the malformation.

At this stage of the surgery we created a plane between the malformation and the brainstem tissue using a diamond knife. By initially using sharp dissection, followed by spreading motions of the bipolar forceps **(2:44)**, we created a plane. Another surgical maneuver which we found very useful was to apply gentle bipolar coagulation at the malformation’s surface, at a location away from the brainstem. Dissection was then carried out rostrally using gentle traction and contratraction.

At this stage we divided the malformation with a microscissor and then removed part of the malformation. We then found a gliotic plane between the brainstem and the vascular lesion and used this to circumferentially dissect along the lesion. Here we again used traction and contratraction technique to develop the plane. It is important to note that coagulation of the normal brainstem tissue should be done to avoid thermal injury to the brainstem. We continued this dissection to develop a plane both caudally and laterally.

We then resected the mass piecemeal and continued the circumferential dissection using microsurgical techniques. The vascular malformation was dissected away at the caudal aspect and a microcotton patty was placed on this aspect of the dissection plane.

We emphasize the importance of preserving the developmental venous anomaly **(3:54)**, which traversed the dorsal aspect of the malformation. This should be respected at all times. Only the small venous drainage tributaries should be coagulated and divided. At this stage, we then bisected the malformation to facilitate safer dissection. This prevents excessive traction on the brainstem.

Careful dissection was patiently continued until every last piece of the malformation was removed. Here we see the final view after complete resection of the cavernous malformation **(4:25)**. The SSEP and MEP neuromonitoring responses remained unchanged throughout the case **(4:31)**. We also obtained meticulous hemostasis in the resection cavity. The wound was then closed in layers.

The surgery and postoperative course were uneventful. The postoperative MRI showed gross-total resection of the mass **(4:42)**. Histopathology indicated a cavernous malformation. The patient continued to do well without recurrence at 7 years’ follow-up.
